# P186: Infection control plan management in primary care

**DOI:** 10.1186/2047-2994-2-S1-P186

**Published:** 2013-06-20

**Authors:** I Neves, F Vieira, D Peres, I Devesa, V Alves

**Affiliations:** 1 Infection Control Unit, Unidade Local de Saúde de Matosinhos, Matosinhos, Portugal

## Introduction

There was a significant shift in healthcare delivery from the acute, inpatient hospital setting to a variety of ambulatory and community-based settings. This transition of healthcare has demonstrated the need for understanding and implementation of infection prevention guidance [1] Portuguese National Infection Control Program includes in its objectives the community and ambulatory settings [2,3].

## Objectives

Implementation and monitoring of an Infection Control Plan in a community-based setting (with several units), part of a Local Health Unit (which includes primary, acute and rehabilitation care).

## Methods

Application of Demming cycle (Plan, Do, Check; Act) in a perspective of continuous improvement according to the institution’s Quality Management System [4]. 

## Results

The Infection Control Unit, in collaboration with link professionals (doctor and nurse in each unit), implements the following strategy: (PLAN) Draw an Infection Control Plan approved by management. Example (i): create the conditions for hand hygiene through scheduled audits. Example (ii): monitoring of good practice, identifying performance critical areas. (DO) Implementation of the activities according to Plan schedule. Example (i): audits, with feed-back to health professionals. Example (ii): scheduled visits to identify areas for improvement. (CHECK) Check compliance of Plan objectives. Example (i): degree of implementation of the planned audits. Example (ii): Multidrug-resistant organisms monitoring (epidemiological surveillance system based on laboratory results); (ACT) Implementation of corrective measures to the initial Plan. Example (i): reprogramming audits not conducted. Example (ii): professionals training directed to good practice (based on critical areas identified and surveillance results).

## Conclusion

Application of a strategy based on Demming cycle allows successful implementation and monitoring of an Infection Control Plan in a community based setting. Primary care still needs the development of specific indicators.

## Disclosure of interest

None declared
